# A case of an AIDS patient with *Cryptococcus neoformans* infection

**DOI:** 10.11604/pamj.2020.36.88.20406

**Published:** 2020-06-15

**Authors:** Bramantono Bramantono, Ahmad Danial, Usman Hadi

**Affiliations:** 1 Tropical and Infectious Disease Division, Department of Internal Medicine, Faculty of Medicine, Universitas Airlangga, Dr. Soetomo Teaching Hospital, Surabaya, East Java, Indonesia,; 2 Department of Internal Medicine, Faculty of Medicine, Universitas Airlangga, Dr. Soetomo Teaching Hospital, Surabaya, East Java, Indonesia

**Keywords:** *Cryptococcus neoformans*, HIV/AIDS, opportunistic infection

## Abstract

Cryptococcosis is the most common fungal disease in HIV-infected persons. It is known as the AIDS-defining illness for 60-70% of HIV-infected patients. Before antiretroviral therapy (ARV) was discovered, fungal and other opportunistic infections were a major problem for people with advanced HIV/AIDS. Presented here is a case of a 43-year-old man who was newly diagnosed HIV, in which he was admitted due to shortness of breath and decreased consciousness. His clinical symptoms, physical examination, laboratory and radiologic findings indicated a *Cryptococcus neoformans* infection. The patient had received treatment using anti-fungal and ARV that showed a clinical improvement during observation.

## Introduction

Cryptococcosis is the most common fungal infection of the central nervous system and may develop into a space-occupying lesion, meningitis, or meningoencephalitis. In addition, cryptococcosis is the most common fungal disease in HIV-infected persons, in which it is known as the AIDS-defining illness for 60-70% of HIV-infected patients [1]. Before antiretroviral therapy was discovered, fungal and other opportunistic infections were a major problem for people with advanced HIV/AIDS. Since then, the numbers of fungal infections and deaths due to fungal infections in advanced HIV/AIDS patients have decreased substantially in the US and other developed countries. One study showed that the incidence of cryptococcosis in AIDS patients in the US decreased by approximately 90% in the 1990 [2,3]. The decrease in opportunistic infections is primarily due to earlier diagnosis of HIV and initiation of antiretroviral therapy (ART) that prevent HIV patients from reaching the stage where their immune systems expose them to most vulnerable to fungal and other infections. However, fungal diseases, particularly cryptococcosis, are still a concern for HIV/AIDS patients in the US [3].

*Cryptococcus neoformans* is a 4-6 microns round-to-oval encapsulated yeast, 3 yeast with a surrounding polysaccharide capsule ranging from 1 to >30 microns in size when cultivated in the laboratory. One study demonstrated that *Cryptococcus neoformans* could be isolated from the nasopharynx of approximately 50% of AIDS patients with cryptococcosis, whereas *Cryptococcus neoformans* was not isolated from AIDS patients without cryptococcosis, using inhalation as a mode of insertion. Although complement-mediated phagocytosis is the primary initial defense against cryptococcal invasion, the absence of an intact cell-mediated response results in ineffective ingestion that kills the organism, leading to dissemination and increased cryptococcal burden. Given its antiphagocytic properties, the polysaccharide capsule, composed mainly of glucuronoxylomannan, is thought to be the organism's primary virulence factor. The exopolysaccharides of the capsule may contribute to virulence by suppressing the immune response, inhibiting leukocyte migration, and enhancing HIV replication [1].

## Case study

**Figure 1:**
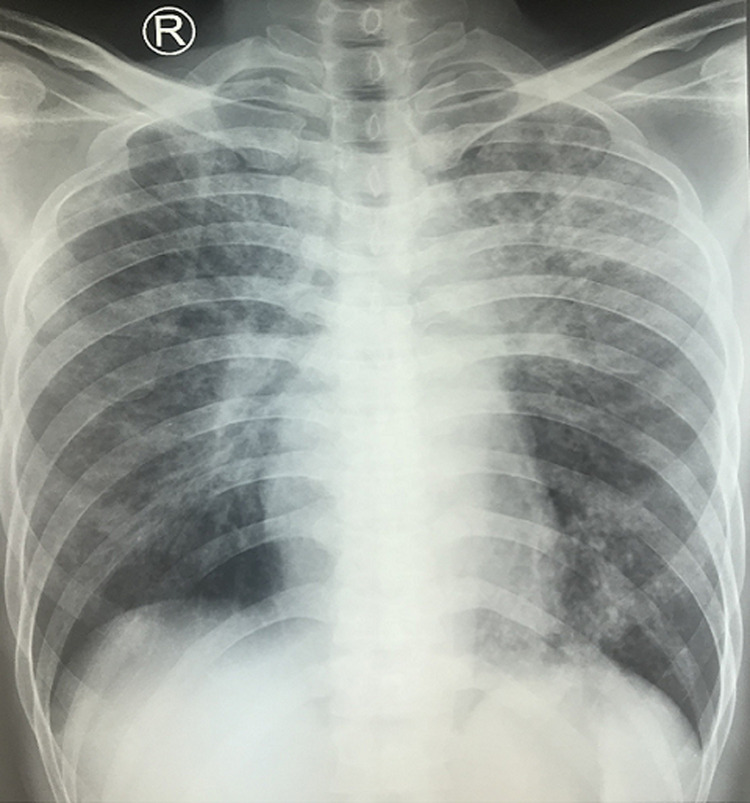
patient's chest X-ray

A 43-year-old male patient was admitted to the emergency department with a complaint of shortness of breath since 2 weeks prior to admission. Shortness of breath was accompanied with cough. Other complains include fever and decreased appetite since 4 months ago due to oral ulcer. He was newly diagnosed with HIV in the previous hospital 2 weeks before being admitted to RSUD Dr. Soetomo Surabaya. From the physical examination, it was found that the patient was somnolent, in which he scored 13 on the Glasgow coma scale and Rhonchi were found in both of his hemithoraces. The chest X-ray showed reticulogranular pattern in both hemithoraces. Trachea in the middle, left and right costophrenicus angle were sharp, soft tissue and visualized bone did not show abnormalities (Figure 1). Head computed tomography (CT) was performed and showed no hypodense/hyperdense lesion in the parenchymal brain. No contrast enhancement were found. Sulcus and gyrus seemed to be normal. The ventricular system and cysterna were normal. Pons and cerebellum were normal. There was no apparent abnormal calcification. No midline deviation was found.

**Figure 2:**
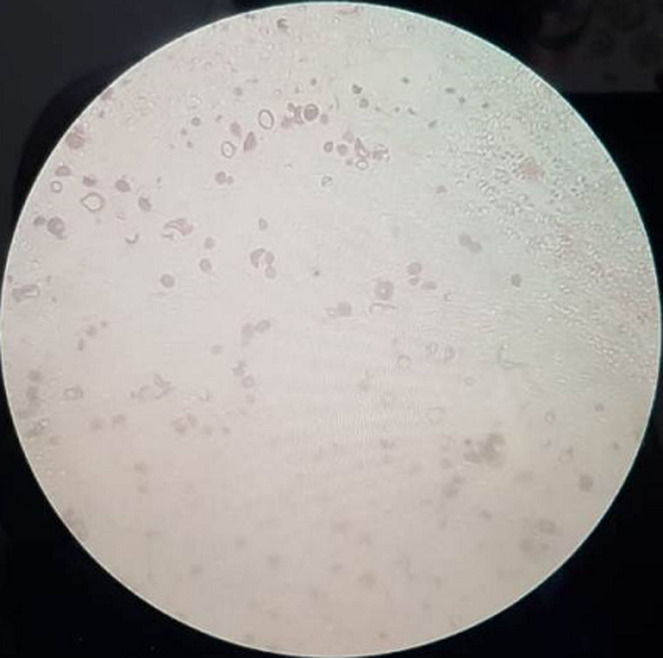
microbiology result

From the sputum and blood culture we found Gram-positive cocci of *Staphylococcus hominis* and yeast cell of *Cryptococcus neoformans* (Figure 2). Blood culture of yeast cell in the form of *Cryptococcus neoformans* is BTA negative. Based on his clinical symptoms, head CT result and blood culture, a diagnosis of *Cryptococcus neoformans* in AIDS was made and the patient received fluconazole treatment at the dose of 750 mg/day and was given ARV therapy with duviral and neviral twice daily. After 2 weeks of treatments, a clinical improvement was obtained and patient was discharged.

## Discussion

*Cryptococcus neoformans* is distinguished from other yeasts by its ability to assimilate urea and it possesses membrane-bound phenoloxidase enzymes, which are able to convert phenolic compounds into melanin as demonstrated by certain agars, such as birdseed agar. It is postulated that *Cryptococcus* has a propensity to invade the central nervous system (CNS) due to its ability to synthesize melanin from catecholamines that are present in this tissue in large concentrations. Melanin production is rarely seen in other *Cryptococcus* species except than *Cryptococcus neoformans*, which allows birdseed agar to be used as a valuable laboratory screening tool [4]. The portal of entry of *Cryptococcus* is usually through inhalation of spores or desiccated yeast cells from the environment. Deposition in the alveoli produces an asymptomatic infection that is either cleared or controlled by a strong cell-mediated immune response leading to a dormant latent infection with development of granuloma in the lungs or hilar lymph nodes. In immunocompromised patients, the fungus can spread from the lungs to the central nervous system and cause meningoencephalitis that is fatal if left untreated.

Once *Cryptococcus neoformans* enters the circulatory system, it must survive in order to disseminate, in which macrophage can be a vessel for dissemination and distribution through the host; when crossing the blood brain barrier (BBB) it is probably mediated by transcytosis (i.e. transcellular penetration through microvascular endothelial cells) and there is evidence that the capsule is involved [1,3]. Central nervous system (CNS) infections is increasing among the rapid grow of HIV-infected patients [5]. *Cryptococcus* spp. tend to invade the CNS, thus leading to life-threatening condition which expose patients to an acute, subacute or chronic meningitis or meningoencephalitis. Signs and symptoms include headache, nausea, fever, altered consciousness, cranial neuropathy, memory loss, confusion, signs of meningeal irritation, nausea and vomiting, seizures, visual and hearing impairment and coma. Signs associated with poor outcome include <15 in Glasgow coma scale (GCS), neurological impairment which involve confusion, abnormal mental state and lethargy, positive blood culture and high CSF cryptococcal antigen (CrAg).

Patients with cryptococcosis may be asymptomatic. Any patients with HIV infection presenting with subacute or chronic headache, particularly those who are CD4-deplete, should be investigated for CM. Lumbar puncture should be performed to measure opening pressure, cerebrospinal fluid (CSF) cell counts, biochemistry culture, CSF CrAg, Gram stain and India ink, along with CrAg serum, blood culture and chest X-ray. CT or magnetic resonance imaging (MRI) brain scans are helpful in assessing cryptococcomas, meningeal inflammation, vasculitis and ventricular compression [6,7]. Amount of microbial infections in HIV patients significantly correlates with decreased CD4 cells [8,9]. Regardless of the symptoms, cryptococcal antigen serum testing should be performed in patients with newly diagnosed HIV - particularly those with advanced HIV and patients from countries where *Cryptococcus* spp. is endemic. The new lateral flow assay (LFA) for measuring cryptococcal antigen designed as a point-of-care test has performed well on serum and CSF (>95% sensitivity and 100% specificity), thus it is comparable to the previously used latex agglutination test.

The LFA assay is currently being used in a number of laboratories in Australia. A positive cryptococcal antigen serum test should trigger investigations for cryptococcal dissemination which can be seen from chest X-Ray +/- CT chest, blood culture, a thorough skin examination and a lumbar puncture to diagnose CM [10]. The treatment for meningitis or other forms of invasive cryptococcal disease is divided into a two-week induction phase, followed by an eight-week consolidation phase and then a prolonged maintenance phase thereafter. An important distinction is made in the guidelines to differentiate treatment of CNS versus non-CNS disease, therefore, a lumbar puncture should be performed among immunocompromised patients with cryptococcal disease to control meningitis. The recommended initial management of cryptococcal meningitis in patients with HIV consists of the rapidly fungicidal regimen of amphotericin B (0.7 to 1 mg/kg/day) plus flucytosine (100 mg/kg/day). Clinical outcomes were improved in AIDS patients with cryptococcal meningitis in South Africa when the induction phase included the combination of flucytosine and amphotericin B, versus amphotericin B alone.

Thirty six studies have shown that amphotericin B (0.7 mg/kg/day) plus flucytosine (100 mg/kg/day) was found to be more rapidly fungicidal and have lower risk of mycological failure at two weeks than amphotericin B alone or in combination with fluconazole [7,10]. Other study mentions that this fungal can be defeated by actinomycetes compound products [11]. Regarding the appropriate dose of amphotericin B, a study compared two different doses (0.7 mg/kg/day versus 1 mg/kg/day) in a group of HIV patients with cryptococcal meningitis in South Africa; both groups were also treated with flucytosine for 14 days. The higher the dose of amphotericin B, the more rapidly fungicidal it is. While there was no difference in mortality in this study, it has been shown that more rapid clearance of infection correlates with improved clinical outcomes.

Improvement in outcomes for patients with cryptococcal meningitis depends not only upon the choice of initial antifungal therapy, but also appropriate management of elevated intracranial pressure. An increased opening pressure is a poor prognostic indicator in cryptococcal meningitis and is associated with higher CNS fungal burden. Therefore, opening pressures greater than 25 cm of CSF should be treated with serial (e.g. daily) lumbar punctures until the pressure normalizes to less than 20 cm of CSF. One approach is to remove a CSF volume that halves the opening pressure (typically 20-30 ml), especially in extremely high pressures. For recurrent symptoms, repeat lumbar puncture should be performed. While CNS mass lesions or cryptococcomas are uncommon, imaging with CT scan or MRI should be performed prior to lumbar puncture to avoid the risk of brain herniation. Placement of temporary lumbar drain may be necessary if opening pressure cannot be otherwise controlled, particularly if neurologic sequelae are persistent [12].

## Conclusion

From the case of a 43-year-old man newly diagnosed with HIV and opportunistic infection of *Cryptococcus neoformans* that is presented above, it can be seen that mortality in HIV-infected patients with cryptococcus is high. Antiretroviral therapy in HIV-infected patients with cryptococcosis should be delayed up to 5 weeks after the start of antifungal therapy. Integrated therapy of HIV and cryptococcosis that includes antifungal therapy, intracranial pressure management for cryptococcal meningitis and the provision of ARVs to restore immune function are required in order for the treatment to be successful.
